# Systematic Global Analysis of Genes Encoding Protein Phosphatases in *Aspergillus fumigatus*

**DOI:** 10.1534/g3.115.016766

**Published:** 2015-05-05

**Authors:** Lizziane K. Winkelströter, Stephen K. Dolan, Thaila Fernanda dos Reis, Vinícius Leite Pedro Bom, Patrícia Alves de Castro, Daisuke Hagiwara, Raneem Alowni, Gary W. Jones, Sean Doyle, Neil Andrew Brown, Gustavo H. Goldman

**Affiliations:** * Faculdade de Ciências Farmacêuticas de Ribeirão Preto, Universidade de São Paulo, 13083-970 Ribeirão Preto, Brazil; † Department of Biology, Maynooth University, Maynooth, Co. Kildare, Ireland; ‡ Medical Mycology Research Center, Chiba University, Chiba 260-8673, Japan; § National Laboratory of Science and Technology of Bioethanol (CTBE), 13083-970 Campinas, Brazil

**Keywords:** *Aspergillus fumigatus*, HOG phosphatases, gliotoxin, iron metabolism, protein phosphatases

## Abstract

*Aspergillus fumigatus* is a fungal pathogen that causes several invasive and noninvasive diseases named aspergillosis. This disease is generally regarded as multifactorial, considering that several pathogenicity determinants are present during the establishment of this illness. It is necessary to obtain an increased knowledge of how, and which, *A. fumigatus* signal transduction pathways are engaged in the regulation of these processes. Protein phosphatases are essential to several signal transduction pathways. We identified 32 phosphatase catalytic subunit-encoding genes in *A. fumigatus*, of which we were able to construct 24 viable deletion mutants. The role of nine phosphatase mutants in the HOG (high osmolarity glycerol response) pathway was evaluated by measuring phosphorylation of the p38 MAPK (SakA) and expression of osmo-dependent genes. We were also able to identify 11 phosphatases involved in iron assimilation, six that are related to gliotoxin resistance, and three implicated in gliotoxin production. These results present the creation of a fundamental resource for the study of signaling in *A. fumigatus* and its implications in the regulation of pathogenicity determinants and virulence in this important pathogen.

*Aspergillus fumigatus* is a filamentous fungus that is able to live in the soil and is capable of causing a wide variety of noninvasive and invasive diseases in mammalian hosts, termed aspergillosis (Greenberger 2002; Dagenais and Keller 2009). One of these human diseases, invasive aspergillosis (IA), has a high frequency of mortality in immunocompromised patients. Aspergillosis is considered a multifactorial disease, because several pathogenicity determinants are required for the establishment of infection. The main factors are hypoxia stress resistance, iron assimilation, gliotoxin production (depending on the immune status of the host), and thermophily (Wezensky and Cramer 2011; Schrettl and Haas 2011; Hartmann *et al.* 2011; Carberry *et al.* 2012; Grahl *et al.* 2012; Scharf *et al.* 2012; Moore 2013; Ding *et al.* 2014; Chotirmall *et al.* 2014; Haas 2014). To effectively combat this life-threatening disease, it is essential to identify and understand cellular mechanisms of how these pathogenicity determinants are coordinated and which signaling molecules are essential for these virulence programs.

Protein kinases and phosphatases are responsible for regulating the continuous equilibrium between protein phosphorylation and dephosphorylation states. The addition or subtraction of phosphate residues by the respective enzymes occurs at specific amino acids such as serine, threonine, and tyrosine residues. Nucleophilic attack of the phosphate ester moiety is the main mechanism of phosphate dephosphorylation (Sanvoisin and Gani 2001; Williams 2004). There are two main families of phosphatases: the serine/threonine (S/T) protein phosphatases and the protein tyrosine phosphatases (PTP) (Pao *et al.* 2007; Shi 2009). S/T phosphatases are made of three subfamilies: phosphoprotein phosphatases (PPPs), metal-dependent protein phosphatases (PPMs), and aspartate-based protein phosphatases, comprising the transcription factor IIF–interacting C-terminal domain phosphatase (FCP/SCP) and haloacid dehalogenase (HAD) classes (Shi 2009; Zhang *et al.* 2010). PTPs are classified into classical protein-tyrosine phosphatases (PTPs), dual-specificity phosphatases (DSPs), low-molecular-weight phosphatases (LMW-PTP), and the CDC25 class phosphatases (Andersen *et al.* 2001; Pao *et al.* 2007; Moorhead *et al.* 2007).

Filamentous fungal phosphatases have been characterized in more detail in *A. nidulans* and *Neurospora crassa* (Son and Osmani 2009; Ghosh *et al.* 2014). In *A. nidulans*, 28 protein phosphatase catalytic subunit genes were identified and systematic deletion analysis identified four essential phosphatases and four required for normal growth (Son and Osmani 2009). The authors have identified several phosphatases involved in different aspects of cell division and mitosis. However, they have not performed an extensive phenotypic analysis of these mutants. More recently, Brown *et al.* (2013) have shown seven of these phosphatases as being involved in cellulase (and in some cases also hemicellulase) production in *A. nidulans*. Subsequently, Assis *et al.* (2015) identified seven other *A. nidulans* phosphatases involved in the regulation of cell cycle, development, and metabolism in response to glucose and alternative carbon sources. *N. crassa* genome encodes catalytic subunits for 30 protein phosphatase genes (Ghosh *et al.* 2014). These authors have characterized phenotypically in detail this null phosphatase collection by demonstrating that 91% of the mutants had defects during growth or asexual development or sexual development, whereas 29% have phenotypes in all three traits. Additionally, chemical sensitivity phenotypes were observed for 17 phosphatase null mutants and nine potential candidates for regulators of the p38 mitogen-activated protein kinase (MAPK) were identified. They have also recognized a phosphatase as a regulator of *N. crassa* female sexual development and *Δcsp-1* and *Δcsp-2*, as important for regulation of conidiation and the circadian clock, respectively (Ghosh *et al.* 2014).

Previously we have identified 32 genes encoding catalytic subunits of protein phosphatases in the *A. fumigatus* genome (Winkelströter *et al.* 2015). Here, we further investigate the functions of protein phosphatases in *A. fumigatus* by generating a null mutant collection for the phosphatase catalytic subunit encoding genes. We were able to construct 24 viable phosphatase null mutants and their growth defects were analyzed, showing that the phosphatase mutants had a great deal of functional redundancy. Several phosphatase mutants had altered sensitivity to cell wall–damaging agents and oxidative and unfolded protein response, stressing chemicals, in addition to geldanamycin (GEL), a heat shock protein 90 (Hsp90) inhibitor. Subsequently, a group of protein phosphatases that possibly played a role in the HOG (high osmolarity glycerol response) pathway was assessed by measuring phosphorylation of the p38 MAPK (SakA) and the expression of osmo-dependent genes. In addition, several phosphatases were shown to be involved in the regulation of virulence factors, including iron assimilation and gliotoxin production/resistance. The phosphatase null mutant collection and these results provide a resource to dissect the signaling pathways and mechanisms involved in regulating virulence and stress tolerance in *A. fumigatus*. This deeper understanding of how virulence mechanisms are coordinated will have both biotechnological and biomedical implications.

## Materials and Methods

### Strains, media, and growth conditions

The *A. fumigatus* parental strains used in this study were CEA17 (control strain) and CEA17-80 (*ku80*^-^; *pyrG*^-^_;_ this strain was used as a recipient strain for the deletion of all phosphatase genes), and Af293 (the parental strain for *ΔsakA*, *ΔmpkC*, and *ΔsakA ΔmpkC*). Media were of two basic types: a complete medium with three variants, YAG (2% glucose, 0.5% yeast extract, 2% agar, trace elements), YUU (YAG supplemented with 1.2 g/liter each of uracil and uridine), and liquid YG or YUU medium of the same compositions (but without agar), and a modified minimal medium (MM: 1% glucose, original high nitrate salts, trace elements, 2% agar, pH 6.5) was also used. Trace elements, vitamins, and nitrate salts are described by Käfer (1977). Expression of genes under the control of *niiA* promoter was regulated by nitrate source: repression on a modified minimal medium (MMM: 1% w/v glucose, 2% w/v agar) plus ammonium tartrate (50 mM) and induction on AMM plus sodium nitrate (10 mM). Strains were grown at 37° unless indicated otherwise. For the experiments of iron starvation, we have grown the strains in MM for 24 hr at 37° and transferred the mycelia to AMM (glucose 1%, salt solution without FeSO_4_0.7H_2_O, and sodium nitrate 70 mM) plus BPS 200 µM [Bathophenanthrolinedisulfonic acid (4,7-diphenyl-1,10-phenanthrolinedisulfonic acid)] and 3-(2-pyridyl)-5,6-bis(4-phenylsulfonic acid)-1,2,4-triazine (ferrozine) 300 µM for 1 or 2 hr at 37°. For the experiments of iron excess, the strains were grown in AMM medium plus BPS 200 µM and ferrozine 300 µM for 24 hr and transferred to AMM plus FeSO_4_0.7H_2_O 200 µM for 1 or 2 hr at 37°. The *A. fumigatus* phosphatase mutants constructed in this study are presented in Table 1. The *A. fumigatus* MAP kinase mutants *ΔsakA*, *ΔmpkC*, *ΔmpkC ΔsakA*, and *ΔmpkA* were constructed by Hagiwara *et al.* (2013, 2014) and Valiante *et al.* (2008), respectively.

**Table 1:** *Aspergillus fumigatus* phosphatase gene families

Family^a^	Subfamily^b^	Class/Domain^c^	*A. fumigatus* ID Genes	*A. fumigatus* Proteins	Effect of the Deletion on *A. fumigatus*	*A. nidulans* Genes	*S. cerevisiae* Protein
S/T	PPP	PP2Ac	Afu5g12010	PphA	Viable	AN0103	Pph3p
S/T	PPP	PP2Ac	Afu5g11370	PpgA	ND^d^	AN0164	Ppg1p
S/T	PPP	PP2Ac	Afu1g04950	GlcA	Lethal	AN0410 *bimG*	Glc7p
S/T	PPP	PP2Ac	Afu6g11470	SitA	Viable	AN0504 *sitA*	Sit4P
S/T	PPP	PP2Ac	Afu2g03950	PpzA	Viable	AN3793	Ppz1p
S/T	PPP	PP2Ac	Afu6g10830	PphB	Lethal	AN6391 *pphA*	Pph21p
S/T	PPP	PP2Ac	Afu5g06700	PptA	Viable	AN10281	Ppt1p
S/T	PPP	PP2Bc	Afu5g09360	CalA/CnaA	Viable	AN8820 *cnaA*	Cmp2p
S/T	PPM	PP2Cc	Afu1g15800	PtcA	Viable	AN0914	Ptc6p
S/T	PPM	PP2Cc	Afu1g09280	PtcB	Viable	AN1358	Ptc2p
S/T	PPM	PP2Cc	Afu8g04580	PpmA	Viable	AN1467	−/−
S/T	PPM	PP2Cc	Afu5g13740,	PtcD,	Both viable	AN2472	Ptc2p
			Afu2g03890	PtcE			
S/T	PPM	PP2Cc	Afu1g06860	PtcF	Viable	AN5722	Ptc5p
S/T	PPM	PP2Cc	Afu5g13340	PtcG	Viable	AN6892	Ptc1p
S/T	PPM	PP2Cc	Afu4g00720	PtcH	Viable	AN2472	Ptc1p
S/T	Asp-based	HAD	Afu1g09460	NemA	Viable	AN1343	Nem1p
S/T	Asp-based	HAD	Afu3g11410	FcpA	Lethal	AN2902	Fcp1p
S/T	Asp-based	HAD	Afu1g04790	PsrA	Viable	AN10077	Psr1p
PTP	Dual-specificity	DSPc	Afu5g11690	PpsA	Viable	AN0129	−/−
PTP	Dual-specificity	DSPc	Afu4g07080	DspC	Lethal	AN4419	−/−
PTP	Dual-specificity	DSPc	Afu2g02760	DspD	Viable	AN4544	−/−
PTP	Dual-specificity	DSPc	Afu3g12250	CdcA	Viable	AN5057	Cdc14p
PTP	Dual-specificity	DSPc	Afu1g13040	DspA	Viable	AN10138	−/−
PTP	Dual-specificity	DSPc	Afu1g03540	DspB	Viable	AN4057	−/−
PTP	Classical	PTPc	Afu3g10970	PtpB	Viable	AN4896	Ptp1p
PTP	Classical	PTPc	Afu4g04710	PypA	Viable	AN6982	Ptp2p
PTP	LMW-PTP	LMWPc	Afu2g01880	LtpA	Viable	AN10570	Ltp1p/Yvh1p
SSU72	SSU72	SSU72	Afu2g03760	SsuA	ND^d^	AN3810	Ssu72p
PTP	CDC-25 type	CDC25	Afu6g08200	NimT	ND^d^	AN3941 *nimT*	−/−
PTP	—	Y-fosfatase	Afu4g07000	YphA	Viable	AN4426	−/−
PTP	—	Y-fosfatase 3	Afu6g06650	PtyA	Viable	AN5767	Ptc7p

a Family abbreviations: S/T, serine/threonine; PTP, protein tyrosine phosphatase.

b Subfamily abbreviations: PPP, phosphoprotein phosphatase; PPM, Mg_2+_ or Mn_2+_-dependent protein phosphatase; Asp-based, aspartate-based phosphatase; LMW-PTP, low-molecular-weight protein tyrosine phosphatase; CDC25 type, cell division cycle 25 type; SSU72, C-terminal domain RNA Pol II phosphatase.

c Class/domain abbreviations: PP2Ac, protein phosphatase 2 A catalytic subunit; PP2Bc, protein phosphatase 2B catalytic subunit; PP2Cc, protein phosphatase 2C catalytic subunit; HAD, haloacid dehalogenase; PTPc, protein tyrosine phosphatase catalytic subunit; DSPc, dual-specificity phosphatase catalytic subunit; LMWPc, low-molecular-weight phosphatase catalytic subunit; CDC25, cell division cycle; SSU72, C-terminal domain RNA polymerase II phosphatase; Y-phosphatase 3, tyrosine phosphatase 3.

d Not determined.

### Analysis of siderophore production by reverse-phase high-performance liquid chromatography

For siderophore production analysis, all strains were grown at 37° in AMM according to the method of Pontecorvo *et al.* (1953), with 2% (w/v) glucose and 20 mM glutamine as carbon and nitrogen sources, respectively. Trace elements were not supplemented with iron and all glassware was washed with concentrated HCl to remove free iron. Liquid cultures (50 ml) were grown in 250 ml conical flasks, inoculated with 10^8^ conidia, at 200 rpm and 37° for 24–72 hr. Culture supernatants (triplicate) were ferrated by the addition of FeSO_4_ to a final concentration of 1.5 mM. Intracellular ferricrocin (FC) analysis was adapted from the work of Szigeti *et al.* (2014). Briefly, mycelia from 24- to 72-hr cultures were harvested and lyophilized and 50 mg mycelia from each strain (duplicate) was added to 750 µl deionized H_2_O in Eppendorf tubes and homogenized by bead beating (10 min) using tungsten beads. Lysates were centrifuged (10,000*g*, 10 min) and supernatants (200 µl) were removed and ferrated by addition of FeSO_4_ to a final concentration of 1.5 mM. All ferrated siderophores were analyzed by reverse-phase high-performance liquid chromatography (RP-HPLC) with DAD (Agilent 1200 system) using a C_18_ RP-HPLC column (Agilent Zorbax Eclipse XDB-C_18_ Semi-Prep; 5 µm, 9.4 × 250 mm) at a flow rate of 2 ml/min. Ferrated fusarinine C (FusC), triacetylfusarinine C (TAFC), and FC were detected at 440 nm. Purified standards of FusC and TAFC were used to determine the respective retention times. The milli absorbance unit (mAU) areas of all siderophores were determined for each sample.

### Analysis of gliotoxin production by LC-MS/MS

For gliotoxin production analysis, all strains were grown at 37° in Czapek-Dox minimal medium. Liquid cultures (50 ml) were conducted in 250-ml conical flasks, inoculated with 10^8^ conidia, at 200 rpm and 37° for 72 hr. Culture supernatants were analyzed by LC-MS/MS on an Agilent 6340 Ion Trap mass spectrometer (Dolan *et al.* 2014). Briefly, samples were organically extracted using chloroform (1:1) and the organic layer was evaporated under vacuum and resuspended in methanol. Samples were diluted 1/10 in 0.1% (v/v) formic acid and spin-filtered (Costar Spin-X) prior to LC-MS analysis; 1 µl of each sample was injected onto a Zorbax 300 SB C_18_ Nano-HPLC Chip (150 mm × 75 μm) with 0.1% (v/v) formic acid at a flow rate of 4 μl/min. Metabolites were eluted using an acetonitrile gradient with a post-run time of 5 min. A commercial standard of gliotoxin (Sigma-Aldrich) was utilized to confirm the retention time and fragmentation pattern of gliotoxin in culture supernatants.

### Phylogenetic analysis

The protein sequences were obtained from the *A. fumigatus* genome database (http://www.aspgd.org) and the *S. cerevisiae* genome database (http://www.yeastgenome.org) (Supporting Information, File S1). The phylogenetic analysis was performed by using MEGA version 6 (Tamura *et al.* 2013). The alignment was performed with CulstalW and manually curated. The evolutionary history was inferred using the Neighbor-Joining method (Saitou and Nei 1987). The percentage of replicate trees in which the associated taxa clustered together in the bootstrap test (500 replicates) is shown next to the branches (Felsenstein 1985). The evolutionary distances were computed using the Poisson correction method and are in the units of the number of amino acid substitutions per site (Zuckerkandl and Pauling 1965). The analysis involved 61 amino acid sequences. All ambiguous positions were removed for each sequence pair. There were a total of 1353 positions in the final dataset.

### DNA manipulations and construction of the *A. fumigatus* mutants

The cassettes for gene replacement were constructed by *in vivo* recombination in *S. cerevisiae* as previously described by Colot *et al.* (2006). Approximately 1.5 kb from the 5′-untranslated region (UTR) and 3′-UTR flanking region of the targeted genes were selected for primer design. The primers 5F and 3R contained a short sequence homologous to the multiple cloning site (MCS) of the pRS426 plasmid. Both the 5- and 3-UTR fragments were PCR-amplified from *A. fumigatus* genomic DNA (gDNA). The *pyrG* inserted into the gene replacement cassettes was amplified from pCDA21 plasmid and was used to generate a marker for prototrophy in the mutant strains. Each fragment along with the *Bam*HI/*Eco*RI cut pRS426 plasmid were transformed into the *S. cerevisiae* strain SC94721 using the lithium acetate method (Schiestl and Gietz 1989). The transformant DNA was extracted according to Goldman *et al.* (2003). The cassette was PCR-amplified from the plasmids utilizing TaKaRa Ex Taq DNA Polymerase (Clontech Takara Bio) and used for *A. fumigatus* transformation. Southern blot was performed as described by Sambrook *et al.* (1989) aiming to demonstrate that the transformation cassettes had integrated homologously at the targeted *A. fumigatus* loci. DNA fragments were labeled with ^32^P-α-dCTP using the RTS Rad Prime DNA labeling System kit (Invitrogen).

The promoter replacement strategy was utilized when the entire gene deletion was not possible. The DNA cassette containing *niiA* was constructed by transformation of the *S. cerevisiae* strain SC9721 with the PCR-amplified fragments of an approximately 1.5-kb 5′ flank, the ORF, and the *pyrG*::*niiA* fragment. The ORF is under the control of the *A. fumigatus niiA* promoter after homologous integration of the translation produces an N-terminal fusion protein. All the transformants were confirmed by PCR using specific primers and by checking if the promoter from *niiA* (encoding a nitrite reductase) is induced by sodium nitrate and repressed by ammonium tartrate (Punt *et al.* 1991). The primers and probes used above are described in Table S1 and Table S2. All the Southern blots, PCRs, and the corresponding strategies to evaluate if the phosphatase genes were either deleted or replaced by *niiA* are shown in Figure S1.

### Phenotypic assays

The phenotypes of the deletion mutants were evaluated either by radial growth or by assessing the initial growth of a droplet of conidia from a serial dilution, at different temperatures, in the presence or absence of oxidative and osmotic stressing agents plus reagents that cause cell wall or DNA damage. Drop out experiments were performed using 5 µl of a 10-fold dilution series starting at a concentration of 2×10^7^ for the wild-type and mutant strains spotted on different growth media and grown for 48 hr at 37°. Additionally, we have performed dry weight experiments by growing different strains for 48 hr at 37° and washing and lyophilizing the mycelia.

### Immunoblot analysis

Detection of SakA phosphorylation by Western blotting was performed as described by Hagiwara *et al.* (2013) with slight modifications. Briefly, *A. fumigatus* conidia were inoculated into liquid YPD (1% yeast extract, 1% polypeptone, and 1% glucose) and cultured for 16 hr prior to addition or not (control) of 1/2 volume 3 M sorbitol (final concentration: 1 M). Mycelia were harvested, frozen in liquid nitrogen, and smashed with 0.5-mm glass beads in protein extraction buffer containing protease inhibitors. The suspension was centrifuged and the supernatant was boiled with an appropriate sample buffer. The protein concentration was determined using a Pierce BCA Protein Assay Kit-Reducing Agent Compatible (Pierce, Rockford, IL).

The same amount (10 µg) of protein was loaded onto NuPAGE Novex Bis-Tris 4–12% gel (Invitrogen). Proteins were separated with NuPAGE system (Invitrogen) and blotted using iBlot gel transfer system (Invitrogen). To detect SakA and phosphorylated SakA proteins, a rabbit polyclonal IgG antibody against Hog1 y-215 (Santa Cruz Biotechnology, Santa Cruz, CA) and a rabbit polyclonal IgG antibody against dually phosphorylated p38 MAPK (Cell Signaling Technology, Beverly, MA) were used, respectively. To detect these signals on blotted membranes, the ECL Prime Western Blotting Detection System (GE Healthcare, Little Chalfont, UK) and LAS1000 (FUJIFILM, Tokyo, Japan) were used.

### RNA and cDNA preparation

Mycelia were harvested and frozen in liquid nitrogen, and total RNA was isolated using the FastRNA Pro Red Kit (MP Biomedicals, Santa Ana, CA). To obtain cDNA pools from the total RNA, the possible contaminating genomic DNA was removed and reverse-transcription was performed using the ReverTra Ace qPCR RT Master Mix with gDNA remover (Toyobo, Osaka, Japan).

### Quantitative real-time RT-PCR

Real-time RT-PCR analysis was performed using the 7300 system (Life Technologies Corporation, Carlsbad, CA) with SYBR Green detection as described previously (Hagiwara *et al.* 2013). Briefly, the Thunderbird SYBR qPCR Mix was used for reaction mixture preparation. The primer sets for the analyses are listed in Table S1. The relative expression ratios were calculated by the ΔCt method. The actin gene was used as a normalization reference for target gene expression level, and wild-type before sorbitol treatment was set as the calibrator in each experiment. Each sample was tested in triplicate.

## Results

### Phenotypic characterization of *A. fumigatus* phosphatase null mutants

Previously, by using a combination of bioinformatics approaches, we were able to identify all the putative protein phosphatases in the *A. fumigatus* genome (Winkelströter *et al.* 2015). This analysis identified 32 *A. fumigatus* phosphatase catalytic subunit encoding genes in accordance with the *A. fumigatus* genome database (www.aspgd.org), and they were named following their S. cerevisiae homologues (Table 1 and Figure S2). These phosphatases were classified as 19 S/T members (8 PPP, 8 PPM, and 3 Asp-based subfamily members), 11 PTP members (6 dual-specificity, 2 classical, 1 LMW-PTP, 1 Cdc25-type, and 1 SSU72), and 2 fungal-specific phosphatases in the PTP family (YphA and PtyA) (Table 1) (Winkelströter *et al.* 2015).

**Figure 1:**
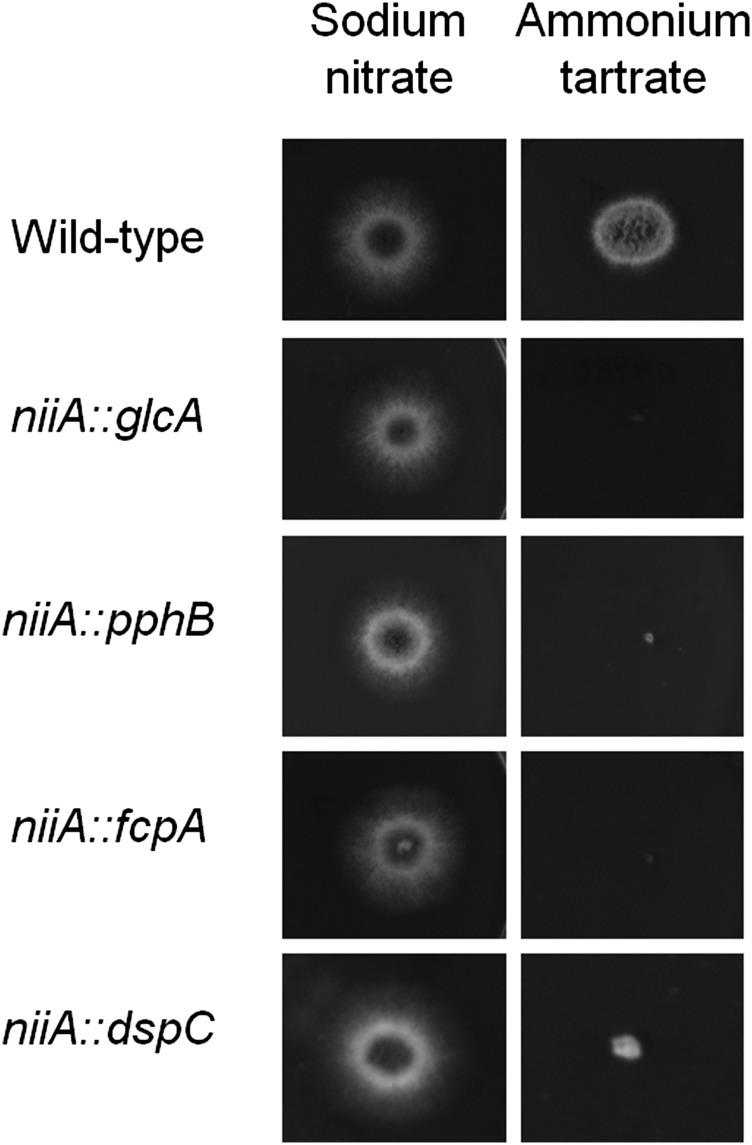
Essential *A. fumigatus* phosphatase encoding genes. The wild-type, *niiA*::*glcA*, *niiA*::*pphB*, *niiA*::*fcpA*, and *niiA*::*dspCA* were grown for 48 hr at 37° on MM+sodium nitrate (induced) and MM+ammonium tartrate (repressed).

To gain a deeper insight into the function of the identified phosphatases, we attempted to construct null mutants for the 31 phosphatase encoding genes (because *A. fumigatus calA* gene encoding the catalytic subunit of calcineurin has already been deleted) (Steinbach *et al.* 2007; Da Silva Ferreira *et al.* 2007). We were able to construct 24 null mutants lacking a single phosphatase (Table 1). The inability to generate null mutants for the remaining seven genes could have been due to the fact that these were essential genes. Thus, conditional mutants were constructed for these genes by replacing the endogenous promoters with the *niiA* promoter (from the *A. fumigatus* nitrite reductase gene). The *niiA* promoter is induced by sodium nitrate and repressed by ammonium tartrate. Four of these genes (*glcA*, *pphB*, *fcpA*, and *dspA*) were shown to be essential (Figure 1). The functionality of the remaining three strains could not be assessed as the construction of null or conditional mutants (by using either *niiA* or *alcA* from the alcohol dehydrogenase gene and promoters) were unsuccessful for *ppgA* (Posas *et al.* 1993), *ssuA* (Sun and Hampsey 1996), and *nimT* (Russell *et al.* 1989). In *A. nidulans*, the homologous *ppgA* and *ssuA* null mutants have reduced fitness (Son and Osmani 2009), whereas the *nimT* homolog, also *nimT*, is an essential *A. nidulans* gene involved in mitosis progression (Son and Osmani 2009). Therefore, these genes are likely to also be essential genes in *A. fumigatus*.

**Figure 2:**
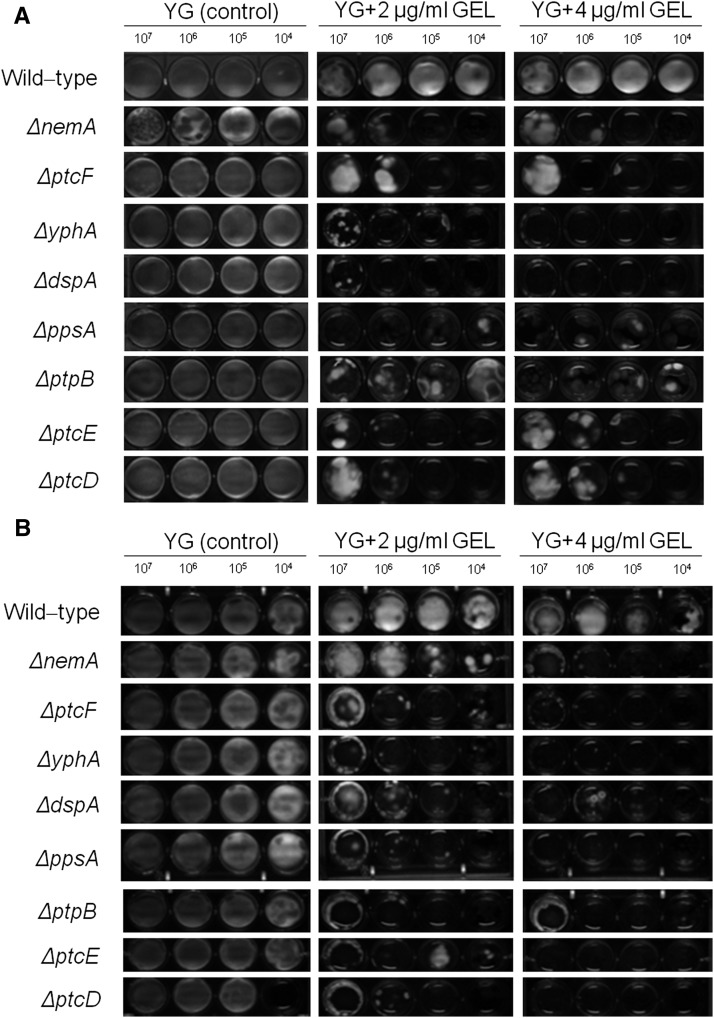
The *A. fumigatus* phosphatase mutants that were sensitive to GEL (Geldanamycin). Ten-fold conidial dilutions (10^7^ to 10^4^) of the wild-type and phosphatase null mutants were grown in MM in the absence or presence of different GEL concentrations for 48 hr at 37° (A) and 30° (B).

**Figure 3:**
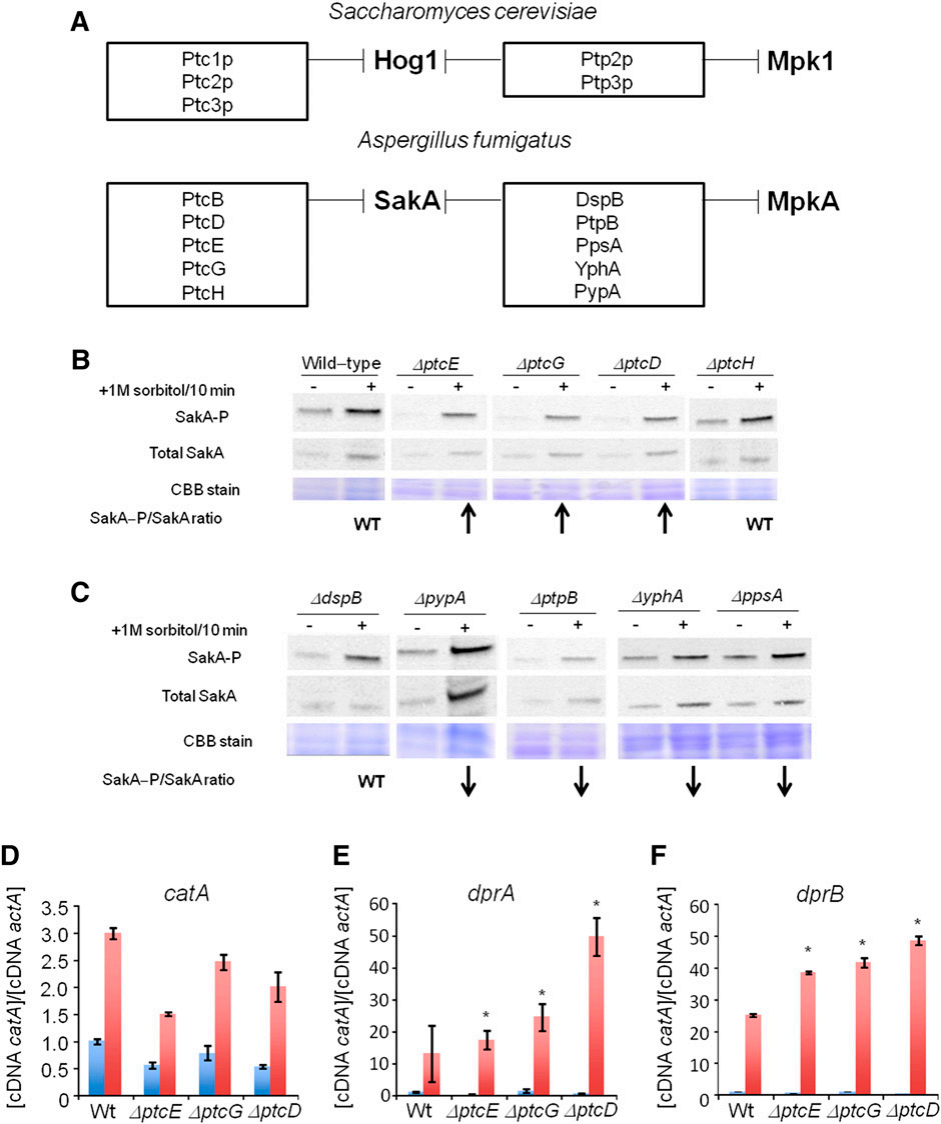
Identification of putative SakA phosphatase null mutants with increased SakA phosphorylation. (A) The putative *A. fumigatus* SakA and dual SakA/MpkA phosphatase homologs. Upper panel shows the *S. cerevisiae* Hog1p and dual Hog1p/Mpk1 phosphatases and the lower panel shows the putative *A. fumigatus* SakA and dual SakA/MpkA phosphatases. These data were based on the phylogenetic analysis shown in Figure S2. (B) and (C) Immunoblot analysis for SakA phosphorylation in response to osmotic stress. The wild-type and the phosphatase null mutants were grown for 18 hr at 37°. Then, sorbitol (1 M final concentration) was not added (control) or added for 10 min. The mycelium was harvested at the indicated times, and total proteins were extracted. Anti-phospho-p38 was used to detect the phosphorylation of SakA, and anti-Hog1p was used to detect the total SakA protein. A Coomassie Brilliant Blue (CBB)-stained gel is shown as a loading control for both gels. Signal intensities were quantified using the Image J software by dividing the intensity of SakA-P/SakA ratio. The experiment was repeated at least three times and a representative blot is shown. The “WT” signifies that the levels of SakA-P/total SakA on osmostress were similar to wild-type, whereas the arrows ↑ and ↓ that correspond to the levels of SakA-P/total SakA on osmostress were higher or lower than the wild-type, respectively. Phosphatase null mutants show higher expression of osmostress-dependent genes. The wild-type and the phosphatase null mutants were grown for 18 hr at 37°. Then, sorbitol (1 M final concentration) was added for 0 (control) and 10 min. The mycelium was harvested at the indicated times, and total RNA was extracted. The relative expression ratios of *catA* (D), *dprA* (E), and *dprB* (F) and *actA* (Afu6g04740, encoding the actin) were calculated by the ΔCt method. The results are the means (± SD) of three biological replicates (*P < 0.001, comparison of the treatments with the time zero control).

**Figure 4:**
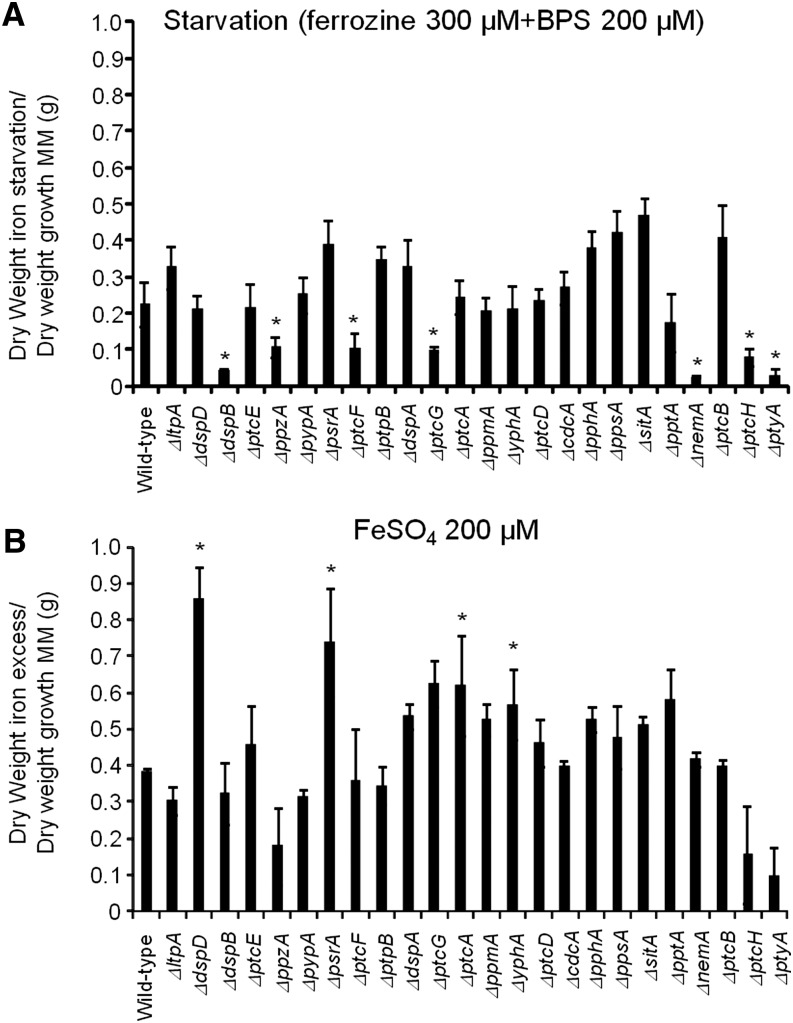
Identification of phosphatase null mutants involved in iron assimilation. The wild-type and the phosphatase null mutants were grown for 48 hr at 37° in minimal medium (MM), in AMM+ferrozine 300 μM+BPS 200 μM (iron starvation), or AMM+200 mM FeSO_4_ (iron excess). After this period, the mycelia were washed with sterile water and dried (*P < 0.001, comparison of the null mutants with the wild-type strain). The results are expressed as (A) dry weight of the strains grown on iron starvation divided by dry weight of the strains grown in MM and (B) dry weight of the strains grown on iron excess divided by dry weight of the strains grown in MM.

**Figure 5:**
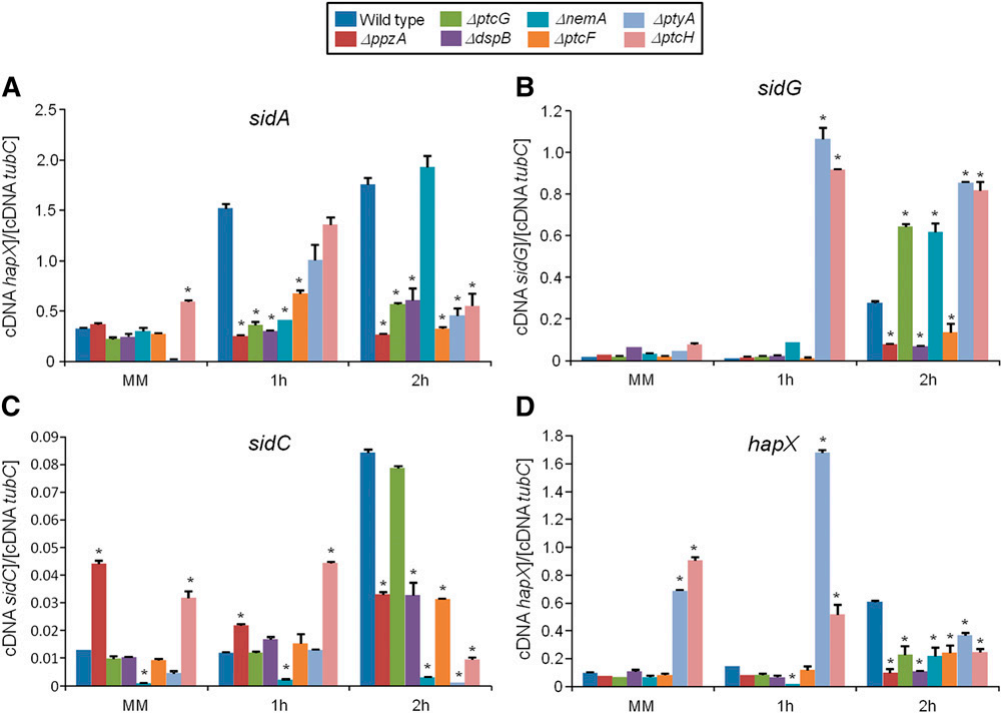
Phosphatase null mutants show altered expression of iron-dependent genes during iron starvation. The wild-type and the phosphatase null mutants were grown for 18 hr at 37° in MM. Then, the mycelia were transferred to AMM+ferrozine 300 μM+BPS 200 μM for 1 hr or 2 hr. The mycelium was harvested at the indicated times, and total RNA was extracted. The absolute quantitation of *sidA* (A), *sidG* (B), *sidC* (C), and *hapX* (D), and the normalizer *tubC*, was determined by a standard curve (*i.e.*, C_T_ values plotted against a logarithm of the DNA copy number). The results are the means (± SD) of three biological replicates (*P < 0.001, comparison of the null mutants with the wild-type strain).

**Figure 6:**
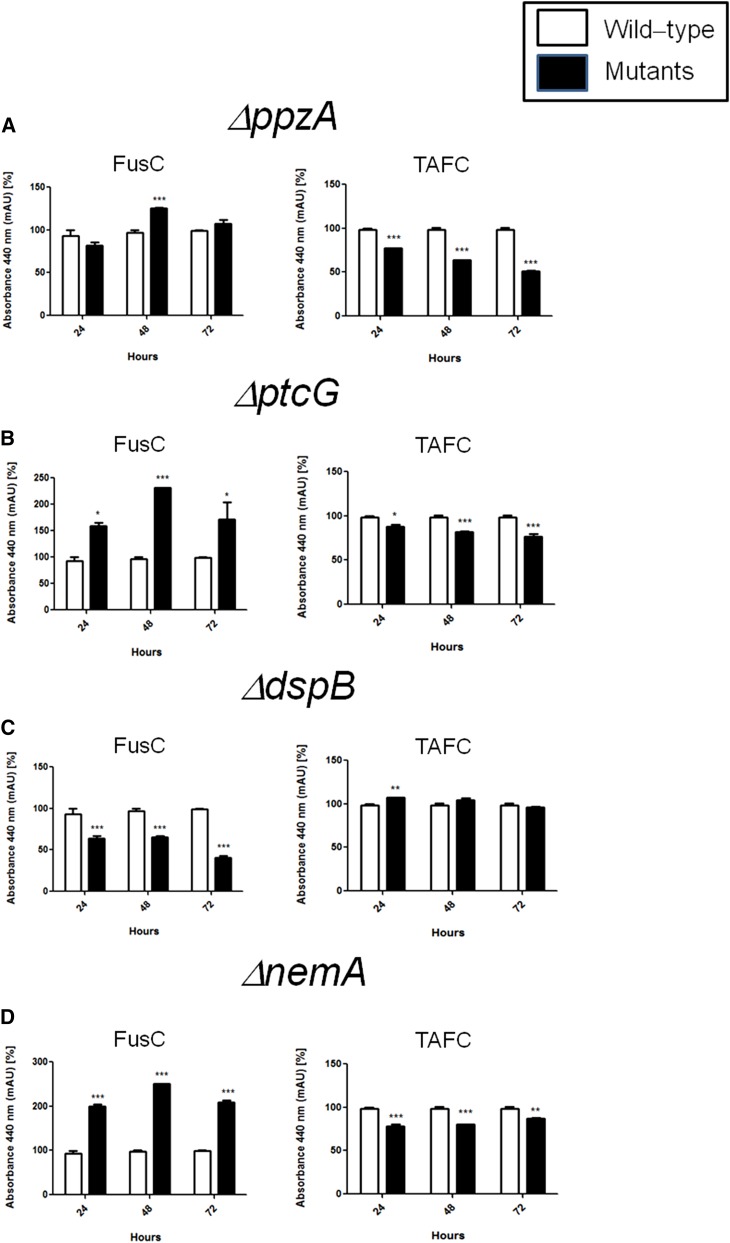
FusC and TAFC production in the wild-type and phosphatase null mutants that have reduced growth during iron starvation. The bars represent the integration areas of the FusC and TAFC peaks identified from culture supernatants analyzed by RP-HPLC (reverse-phase high-performance liquid chromatography). *P < 0.05; **P < 0.01; and ***P < 0.001.

**Figure 7:**
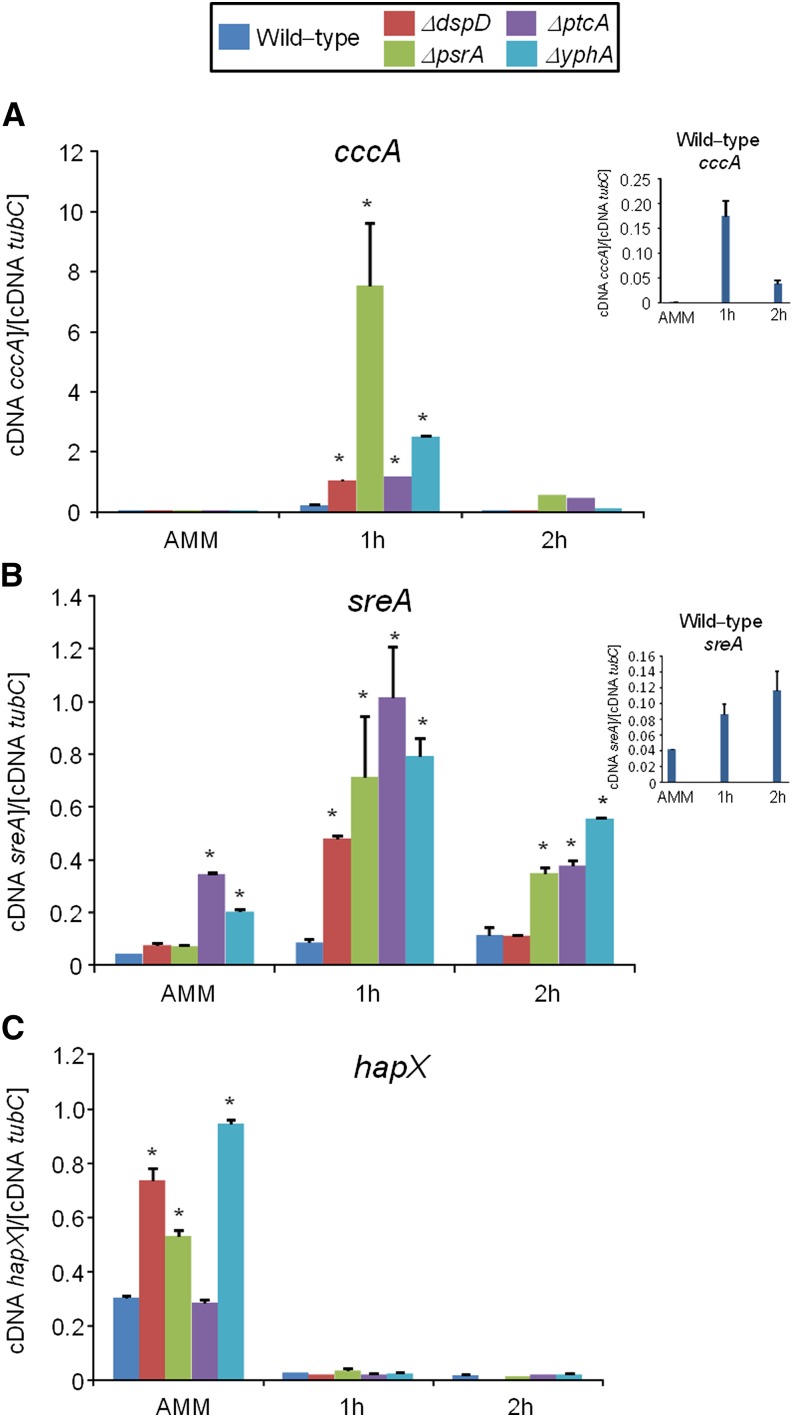
Phosphatase null mutants show altered expression of iron-dependent genes during iron excess. The wild-type and the phosphatase null mutants were grown for 18 hr at 37° in AMM+ferrozine 300 μM+BPS 200 μM. Then, the mycelia were transferred to AMM+200 mM FeSO_4_ for 1 hr or 2 hr. The mycelium was harvested at the indicated times, and total RNA was extracted. The absolute quantitation of *cccA* (A), *sreA* (B), *hapX* (C), and the normalizer *tubC*, was determined by a standard curve (*i.e.*, C_T_ values plotted against a logarithm of the DNA copy number). The insets in (A) and (B) show the results of the wild-type. The results are the means (± SD) of three biological replicates (*P < 0.001, comparison of the null mutants with the wild-type strain).

**Figure 8:**
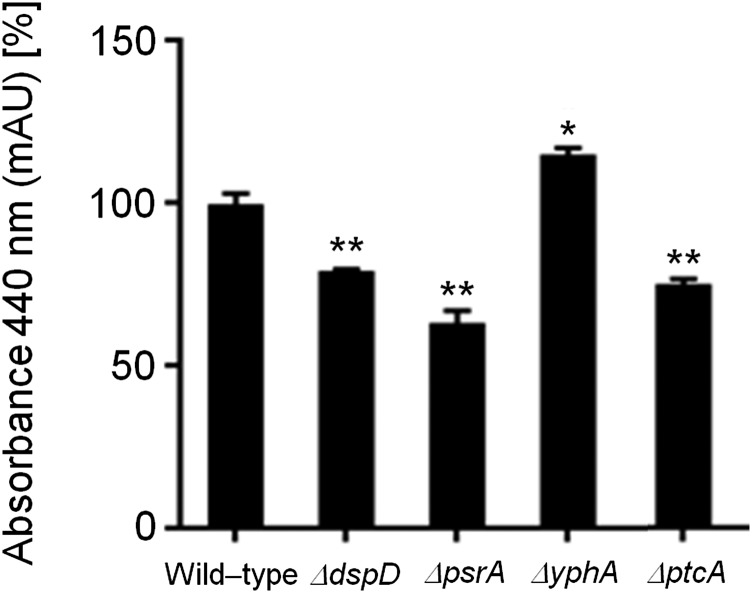
Ferricrocin (FC) production in the wild-type and phosphatase null mutants that have increased growth during iron excess. The bars represent the integration areas of the FC peaks identified from culture supernatants analyzed by RP-HPLC (reverse-phase high-performance liquid chromatography). The asterisks indicate statistical analysis using unpaired *t*-test (*P < 0.05 and **P < 0.01 when compared to the wild-type strain).

**Figure 9:**
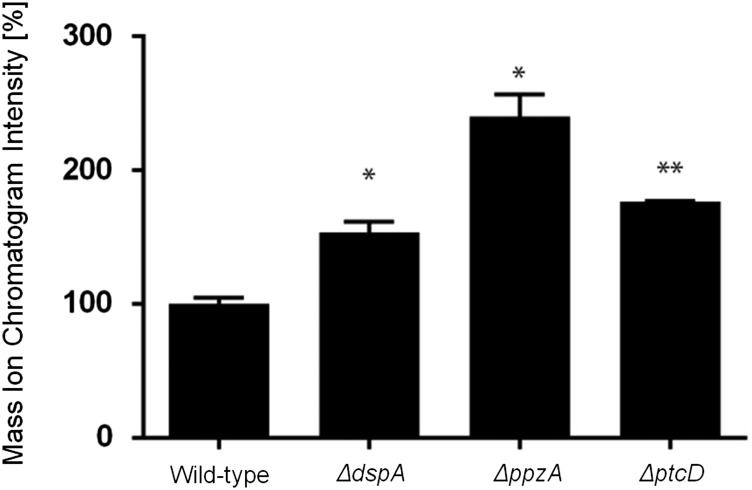
Gliotoxin (GT) detection in *wild-type*, *ΔdspA*, *ΔppzA*, and *ΔptcD*. The quantitative values represent the normalized intensity of GT determined by its diagnostic mass ion chromatograms (m/z 327). The bars represent the mean of three samples and error bars represent SE. The asterisks indicate statistical analysis using unpaired *t*-test (*P < 0.05 and **P < 0.01 when compared to the wild-type strain).

**Figure 10:**
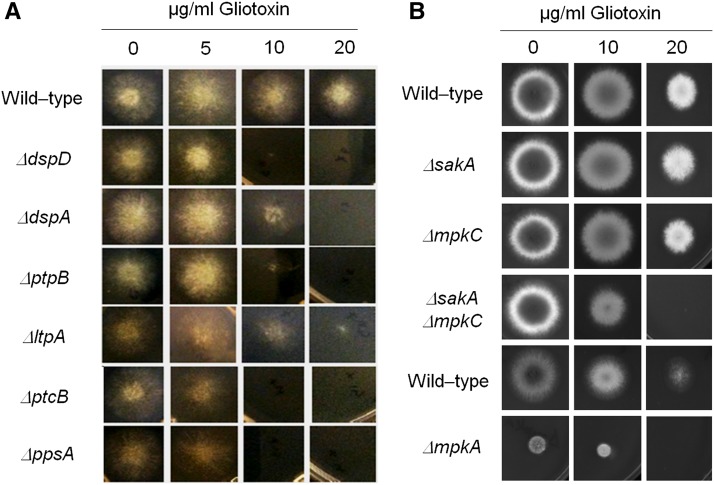
Identification of phosphatase null mutants more sensitive to gliotoxin. The wild-type and the phosphatase null mutants were grown for 72 hr at 37° in MM plus different concentrations of gliotoxin (A). Corresponding wild-type (strains Af293 for *ΔsakA*, *ΔmpkC*, and *ΔsakA ΔmpkC*, and CEA17 for *ΔmpkA*) and MAP kinase null mutants were grown for 72 hr at 37° in MM plus different concentrations of gliotoxin (A).

Growth of the 24 null mutants was compared to the wild-type strain in the following conditions: (1) different temperatures (30°, 37°, and 44°); (2) in media of different nutritional states [complete media (YAG), minimal media (MM), and fetal bovine serum (FBS)]; (3) during calcium starvation [ethylene glycol tetraacetic acid (EGTA)]; (4) for sensitivity to manganese chloride (MnCl_2_); (5) sodium dodecyl sulfate (SDS); (6) oxidative stress (*t*-butyl peroxide, menadione, and paraquat); (7) osmotic stress (NaCl and sorbitol); (8) cell wall–damaging agents [Congo Red (CR) and Calcofluor White (CFW)]; (9) unfolded protein response (UPR) [dithiotreitol (DTT)]; (10) GEL inhibition; (11) iron assimilation; and (12) gliotoxin production/sensitivity (Table 2, Figure 2, Figure 3, Figure 4, Figure 5, Figure 6, Figure 7, Figure 8, Figure 9, Figure 10). No dramatic differences in growth for the phosphatase null mutants were observed in comparison to the wild-type strain (Table 2 and Figure S3), except for the *ΔptcF* and *ΔptcG* mutants that had reduced growth at 44° (Table 2 and Figure S3). In addition, the *ΔppzA*, *ΔnemA*, and *ΔdspD* mutants had reduced conidiation at 44° (Table 2 and Figure S3).

**Table 2:** Growth phenotypes of phosphatase null mutants compared to the wild-type strain

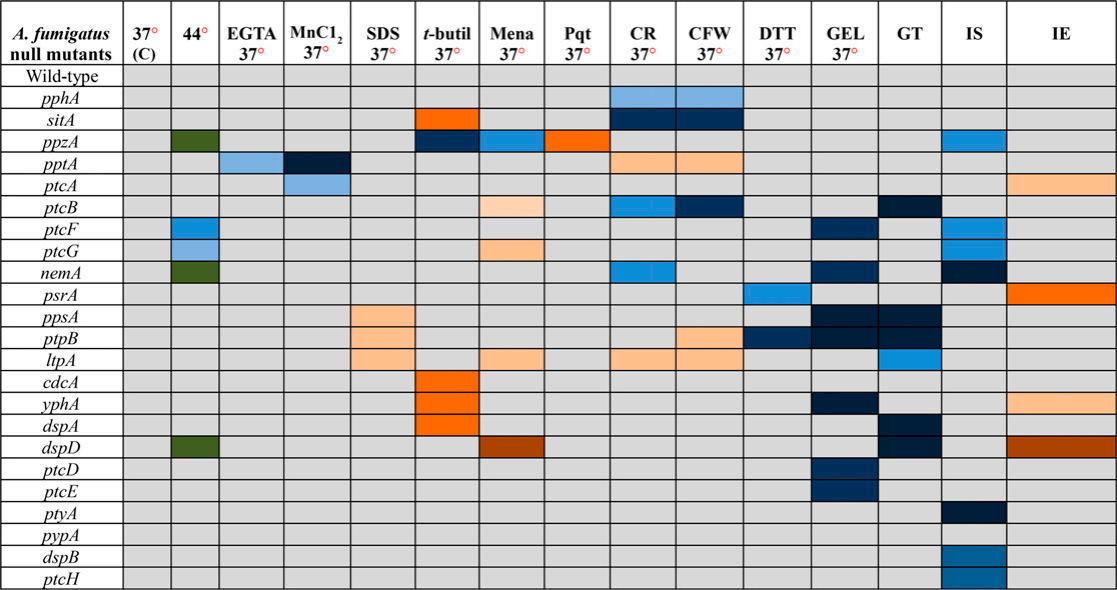

Abbreviations/growth: C, control/solid or liquid medium; EGTA, ethylene glycol tetraacetic acid/solid medium; SDS, sodium dodecyl sulfate/solid medium; *t*-butyl, *t*-butyl peroxide/liquid medium; Mena, menadione/liquid medium; Pqt, paraquat/liquid medium; CR, Congo Red/solid medium; CFW, Calcofluor White/solid medium; DTT, dithiotreitol/liquid medium; GEL, geldanamycin/liquid medium; GT, Gliotoxin/solid medium; IS, iron starvation/liquid medium; and IE, iron excess/liquid medium. Colors are explained in the following chart.
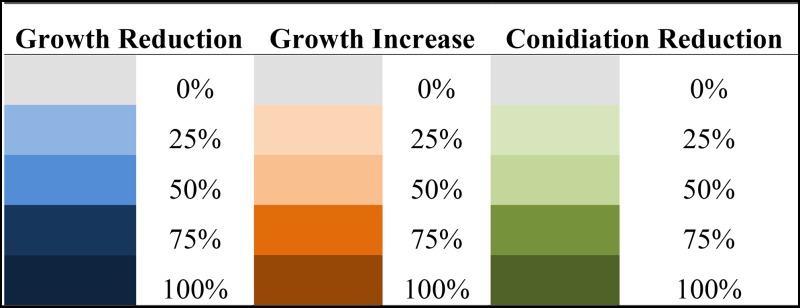

GEL inhibits Hsp90 by binding to its N-terminal ATP domain (Gorska *et al.* 2012). Eight of the phosphatase null mutants were more sensitive to GEL than the wild-type strain when grown at 37° (*ΔnemA*, *ΔptcF*, *ΔyphA*, *ΔdspA*, *ΔppsA*, *ΔptpB*, *ΔptcE*, and *ΔptcD*) (Figure 2A). The same results were observed at 45° (data not shown). To further evaluate if this GEL sensitivity was associated with growth temperature, GEL sensitivity was also assessed at 30° (Figure 2B). Seven null mutants remained sensitive to GEL, whereas the growth of the *ΔnemA* mutant was partially recovered at 30° (Figure 2B). This implied that these phosphatases were important for Hsp90 assembly and/or GEL was affecting other client proteins that interact with these phosphatases. Furthermore, it was possible that NemA participated in a signal transduction pathway associated with Hsp90 and thermo-tolerance. The GEL experiments could not be performed with the *ΔptcB* mutant due to adhesion problems and the fact that this mutant grew poorly in the microtiter plates (Winkelströter *et al.* 2015).

Taken together, these results strongly suggest that *A. fumigatus* phosphatases could have overlapping functions. However, several phosphatases were identified to have specific roles during oxidative and cell wall stressing conditions, whereas others influenced in Hsp90 function.

### *A. fumigatus* HOG response phosphatases

Unexpectedly, none of phosphatase null mutants were very sensitive to osmotic stress either in liquid or in solid media, suggesting the existence of functional redundancy in the *A. fumigatus* osmotic stress pathway. In *S. cerevisiae*, five phosphatases (Ptc1p, Ptc2p, Ptc3p, Ptp2p, and Ptp3p) dephosphorylate Hog1p (Saito and Posas 2012; Brewster and Gustin 2014), negatively regulating the kinase activity of the osmotic stress and cell wall integrity (CWI) pathways (Figure 3A). In *A. fumigatus*, 10 putative homologues of the proteins PtcB, PtcD, PtcE, PtcG, and PtcH (for *S. cerevisiae* Ptc1–Ptc3p) and DspB, PtpB, PpsA, YphA, and PypA (for *S. cerevisiae* Ptp2–Ptp3p) were identified (Figure 3A and Figure S1). Recently, we have identified PtcB as the HOG phosphatase important for *A. fumigatus* virulence (Winkelströter *et al.* 2015). Here, we determine which of the other nine phosphatases were involved in the HOG pathway in *A. fumigatus*. Accordingly, the amount and phosphorylation state of Hog1p homolog, SakA, were determined in the presence and absence of osmotic stress. The phosphorylation level of the SakA protein was determined using the anti-phospho-p38 MAPK (Thr180/Tyr182) and anti-Hog1 (y-215) antibodies (Figure 3, A and B). In the wild-type strain, SakA phosphorylation levels increase approximately twice post-transfer to sorbitol 1 M for 10 min (Figure 3B). Ten minutes was chosen because this was previously shown to be the time point with the highest SakA phosphorylation (Hagiwara *et al.* 2013). The *ΔdspB*, *ΔptcH*, and *ΔppsA* mutants demonstrated levels of SakA induction comparable to the wild-type strain, whereas the *ΔptpB*, *ΔpypA*, and *ΔyphA* mutants did not show any induction (Figure 3, B and C). Thus, the PtpB, PypA, and YphA phosphatases may perform functions important for the SakA phosphorylation. Interestingly, *ΔptcE*, *ΔptcG*, and *ΔptcD* mutants had increased levels of SakA phosphorylation (Figure 3B). Taken together, these results suggest that at least three other phosphatases besides PtcB influence the HOG pathway in *A. fumigatus*.

The genetic markers used to evaluate the induction of the HOG pathway in *A. fumigatus* included *catA* (catalase, Afu6g12180), *dprA* (dehydrin, Afu4g00860), and *dprB* (dehydrin, Afu6g12180). The mRNA accumulation of these genes was determined in the wild-type, *ΔptcE*, *ΔptcG*, and *ΔptcD* strains post-exposure to sorbitol (Figure 3, D–F). Catalase and dehydrin-like proteins play a role in oxidative, osmotic, and pH stress responses, and their expression is dependent on the HOG pathway (Wong Sak Hoi *et al.* 2011). On osmotic stress in the wild-type strain, *catA*, *dprA*, and *dprB* demonstrated an approximately 3-, 10-, and 25-fold increase in mRNA accumulation (Figure 3, D–F). In all the tested phosphatase mutants, *dprA* and *dprB* demonstrated higher mRNA accumulation than the wild-type strain (Figure 3, D–F). In contrast, *catA* mRNA accumulation was comparable to the wild-type in all tested null strains (Figure 3, D–F). These results are in accordance with the observed higher SakA phosphorylation levels, suggesting that the absence of these phosphatases results in an increased activation of the HOG pathway.

PtcB is an *A. fumigatus* HOG phosphatase (Winkelströter *et al.* 2015). Thus, to investigate the impact of the *ΔptcB* mutation on the transcriptional accumulation of *ptcE*, *ptcG*, and *ptcD*, and the subsequent redundancy of the system, the transcriptional regulation of these three phosphatase genes was assessed in the *ΔptcB* mutant after exposure to sorbitol (Figure S4). In the *ΔptcB* mutant, *ptcE*, *ptcG*, and *ptcD* mRNA accumulation was higher than in the wild-type strain (Figure S4). Taken together, these results indicate that PtcB and PtcD, together with PtcB (Winkelströter *et al.* 2015), were the major *A. fumigatus* HOG phosphatases, whereas PtcE and PtcG were minor contributors that may perform a greater role in the absence of either PtcB or PtcD.

### Identification of phosphatases involved in iron assimilation

*A. fumigatus* cannot directly use human iron sources such as heme, ferritin, or transferrin (Schrettl and Haas 2011; Moore 2013; Haas 2014). It utilizes both reductive iron assimilation (RIA) and siderophore (low-molecular-mass ferric iron chelators)-mediated iron uptake during murine infection (Schrettl and Haas 2011; Moore 2013; Haas 2014). Two master transcription factors regulate iron assimilation, HapX (during starvation) and SreA (during iron replete or excess) (Schrettl and Haas 2011; Moore 2013; Haas 2014). *A. fumigatus* makes four types of siderophores. For iron uptake, it secretes fusarinine C (FsC) and triacetylfusarinine C (TAFC) and accumulates ferricrocin (FC) for hyphal and hydroxyferricrocin (HFC) for conidial iron distribution and storage (Schrettl and Haas 2011; Moore 2013; Haas 2014). HapX controls positively the transcription of several genes involved in FsC and TAFC biosynthesis, such as *sidA* (L-ornithine N^5^-oxygenase; the first committed step in siderophore biosynthesis), *sidC* [nonribosomal peptide synthetase (NRPS) involved in ferricrocin siderophore biosynthesis], and *sidG* (fusarinine C acetyltransferase) but represses *sreA*, whereas SreA induces *cccA* (an iron transporter of the vacuolar membrane, involved in vacuolar iron storage) but represses *hapX* (Schrettl and Haas 2011; Moore 2013; Haas 2014).

To identify phosphatases that regulate events involved in iron starvation or excess, we initially grew the wild-type and the phosphatase null mutants in conditions of iron replete, excess (200 mM FeSO_4_), and starvation for 48 hr (Figure 4). All the null phosphatase mutants have biomass growth comparable to the wild-type in iron replete conditions (data not shown). Seven mutants grew significantly less than the wild-type in iron starvation conditions: Δ*ppzA*, Δ*ptcG*, Δ*dspB*, Δ*nemA*, Δ*ptcF*, Δ*ptcH*, and Δ*ptyA* (Figure 4A). Four mutants grew better than the wild-type in iron excess conditions: Δ*dspD*, Δ*psrA*, Δ*ptcA*, and Δ*yphA* (Figure 4B). The wild-type and the mutants were grown for 24 hr in iron replete or iron starvation conditions and then transferred to either iron starving or iron excess conditions for 1 or 2 hr (Figure 5 and Figure 7). As expected, in the wild-type strain during iron starvation conditions, *sidA*, *sidC*, *sidG*, and *hapX* exhibited increased mRNA accumulation (Figure 5, A–D). In contrast, in the seven mutants that were unable to grow during iron starvation conditions, there is a very complex pattern of gene expression. The *sidA* mRNA accumulation is lower in the mutants than in the wild-type strain, except for Δ*ptyA* that has a delayed, but comparable, gene expression to the wild-type (Figure 5A). The *sidG* expression is much higher in Δ*ptyA*, Δ*ptcH*, Δ*ptcG*, and Δ*nemA* than in the wild-type, whereas in the Δ*ppzA*, Δ*dspB*, Δ*ptcF*, and Δ*ptcH* it was lower than in the wild-type (Figure 5B). All the mutants have reduction of *sidC* mRNA, except the Δ*ptcG* mutant, which has an expression pattern similar to the wild-type (Figure 5C). All the mutants have reduced *hapX* mRNA accumulation, except Δ*ptyA* and Δ*ptcH*, which have a much earlier and higher (ΔptyA) accumulation than the wild-type (Figure 5D). We have also evaluated FusC and TAFC production in four of these mutants (randomly selected) (Figure 6). There is an increased and decreased production of FsC and TAFC, respectively, in all four mutants (Figure 6, A–D).

In the wild-type strain during iron excess conditions, the *cccA* and *sreA* have increased mRNA accumulation, whereas *hapX* has decreased mRNA accumulation after 1 and 2 hr (Figure 7, A–C, and insets in Figure 7, A and B). In contrast, most of the four mutants that have increased biomass in iron excess had an increased mRNA accumulation of *cccA* and *sreA*, and much lower *hapX* mRNA accumulation than in the wild-type strain (Figure 7, A–C). Surprisingly, only one of these four mutants, Δ*yphA*, exhibited higher intracellular FC production than the wild-type, whereas the three other mutants have lower FC production than the wild-type (Figure 8).

Taken together, these results strongly suggest that these phosphatases are involved directly or indirectly in post-translational modifications that affect the response to iron assimilation, impacting not only the transcription of several genes involved in the siderophore biosynthesis but also the siderophore production.

### Recognition of phosphatase null mutants with higher gliotoxin production and sensitivity

Gliotoxin is an epidithiodioxopiperazine (ETP)-type fungal toxin that performs an important role in *A. fumigatus* virulence by inducing apoptosis in macrophages and modulating the immune response (Scharf *et al.* 2012). All the phosphatase null mutants produce gliotoxin levels comparable to the wild-type strain, except for the null mutants for *ΔdspA*, *ΔptcD*, and *ΔppzA*, which were able to produce 1.5-, 1.8-, and 2.4-fold more gliotoxin than the wild-type strain (Figure 9, Figure S5, Figure S6). The *ΔdspD*, *ΔdspA*, *ΔptpB*, *ΔltpA*, *ΔptcB*, and *ΔppsA* mutants showed increased sensitivity to gliotoxin than the wild-type strain (Figure 10A). Considering that four of these phosphatases, PtcD, PtpB, PpsA (shown here), and PtcB (Winkelströter *et al.* 2015) are HOG phosphatases (see Figure 3), we decided to investigate the contribution of different *A. fumigatus* MAP kinases to gliotoxin sensitivity (Figure 10B). There are four MAPKs in *A. fumigatus*: (1) MpkA (regulation of CWI signaling and pyomelanin formation); (2) MpkB (mating, putative pheromone signaling); (3) MpkC (regulation of conidium germination); and (4) SakA (the Hog1 ortholog that is involved in osmotic stress, carbon and nitrogen starvation, and regulation of conidium germination) (Xue *et al.* 2004, Reyes *et al.* 2006; May 2008; Valiante *et al.* 2008, 2009). The *ΔsakA*, *ΔmpkC*, *ΔsakA ΔmpkC*, and *ΔmpkA* strains were tested for gliotoxin sensitivity (Figure 10B; note that the parental strain of *ΔmpkA* is Af293, whereas for the other mutants CEA17 is the parental strain). The double mutant *ΔsakA ΔmpkC* was much more sensitive to gliotoxin than the single null mutants and the wild-type strains (Figure 10B). Taken together, these results suggest that SakA and MpkC kinase and phosphatase pathways are redundant and important for gliotoxin resistance.

## Discussion

Protein phosphatases have been portrayed as important in virulence and pathogenicity in several human and pathogenic fungi (Erental *et al.* 2007; Di Stasio *et al.* 2009; Rispail *et al.* 2009; Feng *et al.* 2010; Jiang *et al.* 2011; Ariño *et al.* 2011; Adám *et al.* 2012; Du *et al.*, 2013; Yang *et al.* 2013a,b; Shin *et al.* 2013; Muszkieta *et al.* 2014: Lee *et al.* 2014; Yu *et al.* 2014). The presented study of a collection of *A. fumigatus* protein phosphatase null mutants demonstrates the value of this novel biological resource in dissecting the signaling pathways involved in infection. Subsequently, this investigation revealed the importance of multiple phosphatases in regulating virulence traits, particularly osmotic stress resistance, iron assimilation, and gliotoxin production/sensitivity. We have reconstituted *ΔptpB* (Winkelströter *et al.* 2015), *ΔppzA*, *ΔptcG* (data not shown), and *ΔsitA* (V. L. P. Bom, unpublished data) strains and confirmed that the corresponding phenotypes observed here are only due to the phosphatase mutations. Before our investigation, there were only two previous studies reporting *A. fumigatus* phosphatase PhzA (protein phosphatase Z, here named PpzA) being involved in oxidative stress resistance (Leiter *et al.* 2012; Muszkieta *et al.* 2014) and the HOG phosphatase PtcB (Winkelströter *et al.* 2015).

The *A. fumigatus* genome contains 32 putative protein phosphatase catalytic subunit-encoding genes. However, not all genes could be deleted during the construction of the phosphatase null mutant collection, with phosphatases including 19 serine/threonine and 13 tyrosine phosphatases. Interestingly, fungi do not possess proper tyrosine kinases that phosphorylate tyrosine residues (Borkovich *et al.* 2004; Kosti *et al.* 2010). The current hypothesis for the presence of tyrosine phosphatases in fungal genomes, including *A. fumigatus*, is that tyrosine phosphatases have evolved before tyrosine kinases because serine/threonine kinases can phosphorylate tyrosine residues to a lesser extent, creating a target for the tyrosine phosphatases (Moorhead *et al.* 2007, 2009). Eight *A. fumigatus* phosphatases, PpmA (Afu8g04580), PpsA (Afu5g11690), DspC (Afu4g07080), DspD (Afu2g02760), DspA (Afu1g13040), DspB (Afu1g03540), NimT (Afu6g08200), and YphA (Afu4g07000) showed very low or no identity to other proteins in *S. cerevisiae*, animals, or plants, whereas putative homologues were identified in *A. nidulans* and *N. crassa* (except for PtyA that has no *N. crassa* homologue). This could indicate these phosphatases are fungal-specific. Similarly, in *N. crassa* two PTPs, *pty-5* and *pty-6*, also appear to be fungal-specific phosphatases, whereas disruption of these phosphatases causes defects in fungal-specific traits, such as conidiation and resistance to fludioxonil (Ghosh *et al.* 2014). For the fungal-specific phosphatase mutants in *A. fumigatus*, we observed sensitivity to GEL and SDS (*ΔppsA*), reduced conidiation at 44° and sensitivity to menadione (*ΔdspD*), sensitivity to *t*-butyl (*ΔdspA*), involvement in the cell cycle (*ΔnimT*), and sensitivity to GEL and *t*-butyl (*ΔyphA*) (Table 2). Additional studies are necessary to evaluate the importance of these putative fungal-specific tyrosine phosphatases in filamentous fungi.

Through extensive screening for *in vitro* phenotypes using combinations of numerous compounds and conditions, it was possible to assign specific phenotypes for all the phosphatase null mutants, except *ΔpypA*. Some of these mutants have complex phenotypes, such as sensitivity to cell-damaging agents and reduced growth during iron starvation. This may reflect the complex net of signal transduction pathways that influence these traits and, accordingly, how these phosphatases are engaged in the activation and repression of different protein interactions. Interestingly, none of the phosphatase mutants displayed sensitivity to high osmolarity, suggesting the existence of functional redundancy. It remains to be investigated if the null phosphatase mutants could affect other aspects of the *A. fumigatus* biology, such as sexual cycle or other stages of the life cycle.

The phosphatase null mutant collection was screened using a chemical genomic approach with GEL, an inhibitor of Hsp90 (Gorska *et al.* 2012). The fungal Hsp90 interactome has numerous client proteins such as receptors, protein kinases, and transcription factors (Leach *et al.* 2012). It has been shown that Cdc37p, an Hsp90 co-chaperone, controls the functionality of the Hog1 and Mpk1 cascades in *S. cerevisiae* (Hawle *et al.* 2007; Yang *et al.* 2007), suggesting that Cdc37p acts as a regulator of MAPK signaling. Diezmann *et al.* (2012) used an equivalent chemical genomic screening approach to identify the *C. albicans* Hsp90 interaction network under diverse stress conditions. This study revealed that the chaperone interactome was dependent on the environment and that most of the 226 genetic interactors were important for growth only under specific conditions, suggesting that they operate downstream of Hsp90, as was the case for the MAPK Hog1. Leach *et al.* (2012) have observed that in *C. albicans*, Hsp90 interacts with and downregulates the heat shock transcription factor Hsf1, modulating short-term thermal adaptation, whereas long thermal adaptation depends on cross-talk between the Hog1, Mck1, and Cek1 MAPK cascades. In *C. albicans*, temperature affects the resistance of *C. albicans* to cell wall stresses but not osmotic stress, whereas Hsp90 depletion affects cell wall biogenesis by impairing the activation of its client proteins Mkc1 and Hog1, as well as Cek1 (Leach *et al.* 2012). These results indicate that in *C. albicans* Hsp90 modulates the short-term Hsf1-mediated activation of the classic heat shock response and coordinates this response with the long-term thermal adaptation process via Mkc1-, Hog1-, and Cek1-mediated cell wall remodeling (Leach *et al.* 2012). In *A. fumigatus* neither temperature nor sub-inhibitory concentrations of GEL had a dramatic influence on growth (data not shown). However, eight *A. fumigatus* phosphatase null mutants were sensitive to GEL. Further investigation is required to determine which signal transduction cascades are affected by Hsp90 inhibition in *A. fumigatus*.

*S. cerevisiae* Hog1 kinase is inactivated by the S/T phosphatases Ptc1, Ptc2, and Ptc3, and by the PTP phosphatases Ptp2 and Ptp3 (Jacoby *et al.* 1997; Wurgler-Murphy *et al.* 1997; Warmka *et al.* 2001; Saito and Tatebayashi 2004; Martín *et al.* 2005; Saito and Posas 2012). In *S. cerevisiae* Ptp2 and Ptp3 also inactivate Mpk1 of the CWI pathway (see Figure 4) (Mattison *et al.* 1999; González *et al.* 2006). In *N. crassa* there are nine potential candidates for regulators of the p38 MAPK; among them are *Δpph-8* and *Δpty-3* (Ghosh *et al.* 2014). *A. fumigatus* PtcB, PtcD, PtcE, and PtyA are possible homologues of *N. crassa pph-8* and *pty-3*, respectively. The presented study identified at least three putative Hog1 phosphatases in *A. fumigatus* that seem to have different influences on SakA activation state. Recently, we have identified PtcB as an additional SakA phosphatase (Winkelströter *et al.* 2015). Interestingly, PtcB also had a remarkable effect on the cell surface, conidia and germling adhesion, biofilm formation, and MpkA phosphorylation, suggesting PtcB is also involved in the CWI pathway (ten Cate *et al.* 2009; Cuéllar-Cruz *et al.* 2012). However, we were not able to observe any cell wall damage defects in the putative SakA phosphatases PtcD, PtcE, and PtcG. There are only few reports of *Botrytis cinerea* and *Cryptococcus neoformans* Hog1 phosphatases showing their significance in virulence and pathogenicity (Yang *et al.* 2013a,b; Lee *et al.* 2014), and the importance of *A. fumigatus* PtcD, PtcE, and PtcG on the survival in the host remains to be demonstrated.

We have identified 11 phosphatase null mutants as involved in iron metabolism, by showing their reduced or increased growth during iron starvation or excess, respectively. Accordingly, the mRNA accumulation of several genes related to iron starvation or excess and the siderophore production were anomalous when compared with the parental strain, suggesting these phosphatases are affecting transcriptional programs related to iron metabolism or the activity of enzymes involved in siderophore production. Both SreA and HapX transcription factors appear to be regulated post-translationally by iron (Schrettl and Haas 2011; Moore 2013; Haas 2014). Thus, it is possible these alterations in the transcriptional programs are related to post-translational modifications of these transcriptional master regulators performed by these phosphatases. Siderophore biosynthesis is important both for iron starvation and excess (Schrettl and Haas 2011; Moore 2013; Haas 2014). Their biosynthesis is affected by the available concentrations of the amino acid precursors arginine and ornithine and the ergosterol intermediate mevalonate (Schrettl and Haas 2011; Moore 2013; Haas 2014). Accordingly, it is also possible that the defects observed for these phosphatase mutants are dependent on the dynamics of the amino acid pools or changes in the concentration of mevalonate. However, we were not able to observe any change in the biomass of these mutants when they were grown either in starvation or in excess iron conditions in the presence of eflornithine (an ornithine analog) or lovastatin (an inhibitor of Hgm1, hydromethylglutaryl−CoA reductase, the enzyme that converts hydromethylglutaryl−CoA to mevalonate; data not shown).

Depending on the host immune status, gliotoxin is an important virulence factor for *A. fumigatus*; however, gliotoxin can also inhibit fungal development, including *A. fumigatus* growth (Scharf *et al.* 2012; Chotirmall *et al.* 2014; Carberry *et al.* 2012). The mediation of self-protection of *A. fumigatus* against gliotoxin is performed mainly by GliT, a gliotoxin sulfhydryl oxidase required for gliotoxin biosynthesis (Scharf *et al.* 2012; Chotirmall *et al.* 2014; Carberry *et al.* 2012; Schrettl *et al.* 2010). GliT is able to keep gliotoxin in the sulfur-bridged form, avoiding the generation of reactive oxygen species and of protein conjugates (Scharf *et al.* 2012; Chotirmall *et al.* 2014; Carberry *et al.* 2012). All the phosphatase null mutants produce comparable gliotoxin levels than the wild-type, except for the null mutants for DspA, PtcD, and PpzA that were able to produce between 1.5- and 2.4-fold more gliotoxin than the wild-type strain. We have observed six null phosphatase mutants as more sensitive to gliotoxin than the wild-type. These six phosphatases could participate in post-translational modifications that affect GliT activation or general mechanisms of gliotoxin detoxification. Interestingly, some of these phosphatases are putative MAP kinase phosphatases, such as PtpB, PpsA, PtcB, and PtcD. Consequently, we evaluated the gliotoxin sensitivity of three *A. fumigatus* MAP kinase null mutants. Our results strongly suggest that SakA and MpkC collaborate and are influential in mediating gliotoxin resistance in *A. fumigatus*.

This study presents the creation of an important novel biological resource for the dissection of the signal transduction pathways involved in pathogenicity. The value of this resource was subsequently demonstrated through the identification of the phosphatases responsible for the regulation of the SakA-mediated osmotic stress, iron assimilation, and gliotoxin resistance pathways. The continued investigation of the phosphatase mutant collection will reveal new connections among MAPK cascades and other signaling pathways involved in virulence. This could facilitate the design of novel strategies aiming to control this important disease.
